# Estimating the Health-Related Quality of Life Benefit of Prophylactic Treatment for COVID-19 in Immunocompromised People: A Multimethod Valuation Study

**DOI:** 10.36469/001c.120605

**Published:** 2024-07-24

**Authors:** Katy Gallop, Rebekah Hall, Michael Watt, Daniel Squirrell, Neil Branscombe, Sofie Arnetop, Andrew Lloyd

**Affiliations:** 1 Acaster Lloyd Consulting Ltd, London, United Kingdom; 2 AstraZeneca UK Ltd., London, United Kingdom; 3 AstraZeneca UK Ltd., Cambridge, United Kingdom; 4 AstraZeneca Ltd. Gothenburg, Sweden

**Keywords:** COVID-19, pre-exposure prophylaxis, immunocompromised, health utility values, time trade-off, vignettes

## Abstract

**Background:** Pre-exposure prophylaxis (PrEP) for COVID-19 provides additional protection, beyond vaccines alone, for individuals who are immunocompromised (IC). This may reduce the need for preventative behavioral modification, such as shielding—a behavioral restriction limiting an IC individual to minimize face-to-face interactions and/or crowded places. Therefore, PrEP may improve psychosocial well-being and health-related quality of life (HRQoL) for individuals with IC conditions.

**Objective:** To estimate the potential HRQoL and utility benefit of PrEP for prevention of COVID-19 in individuals with IC conditions who may not have an adequate response of full vaccination (and therefore are at “highest risk” of severe COVID-19) that can be used in future economic evaluations of preventative therapies against COVID-19.

**Methods:** Vignettes describing HRQoL associated with 2 pre-PrEP states (shielding and semi-shielding behavioral restrictions) and a post-PrEP state were developed from a literature review and tested through interviews with clinicians (n = 4) and individuals with IC conditions (n = 10). Vignettes were valued by a general population sample (N = 100) using a visual analog scale (VAS), time trade-off (TTO), and EQ-5D-5L. A sample of individuals with IC conditions (n = 48) valued their current HRQoL and a post-PrEP vignette using VAS and EQ-5D-5L.

**Results:** Individuals with IC conditions reported a mean current EQ-5D-5L score of 0.574, and 0.656 for post-PrEP based on the vignette. PrEP would lead to behavior changes for 75% (30/40) of individuals with IC conditions and an emotional benefit for 93% (37/40) of individuals with IC conditions. Mean values from the general population valuation based on EQ-5D-5L ranged from 0.606 (“shielding”) to 0.932 (“post-PrEP”).

**Conclusion:** This study quantified the expected health state utility benefit of reduced psychosocial burden and behavioral restriction. PrEP would potentially result in a utility gain between 0.082 and 0.326, dependent on valuation approach and expected change in behavioral restrictions, leading to improvements in daily activities and emotional well-being.

## BACKGROUND

As of February 2024, there were over 774 million cases of COVID-19 and 7 million deaths worldwide.^1^ Vaccination against COVID-19 has proved to be highly effective in reducing mortality and morbidity associated with the virus.^2,3^ However, vaccine effectiveness is significantly lower in immunocompromised (IC) groups.^4,5^ A recent study demonstrated that, despite being fully vaccinated (≥3 doses), COVID-19 risk remained elevated across individuals with IC conditions by 1.3-to 13.1-fold for COVID-19 hospitalization and 1.3- to 19.9-fold for COVID-19 deaths compared with individuals without an IC condition.^5^ In England, it is estimated that IC individuals facing increased risk of serious COVID-19 infection account for 0.7% to 3.9% of the population, depending on the definition of IC.^5,6^

Pre-exposure prophylaxis (PrEP) for the prevention of COVID-19 aims to reduce the rate and severity of infection and severity of illness for COVID-19 in individuals with IC conditions for whom vaccination may be contraindicated, or who experience an insufficient immune response to vaccination alone.^7^ In the United Kingdom, despite approval by the Medicines and Healthcare Products Regulatory Agency, no PrEP interventions have been recommended for use by health technology assessment bodies in the UK, including the National Institute for Health and Care Excellence (NICE) and the Scottish Medicines Consortium.^8,9^

When establishing the suitability of PrEP, the primary focus of cost-effectiveness analysis should be to establish the ability of the intervention to reduce the risk of symptomatic COVID-19, hospitalization, long-term effects of COVID-19 (eg, long COVID), and death. However, there may also be benefits to patients’ health-related quality of life (HRQoL) in terms of daily activities, reduced need for social isolation, and an improvement in emotional well-being.^10^ In March 2020, official guidance from the UK government recommended that clinically vulnerable individuals “shield” themselves against COVID-19 by strictly avoiding contact with anyone displaying coronavirus symptoms and staying at home. Despite official shielding guidance ending in April 2021, a study in 2022 reported that IC individuals continue to modify their behavior due to COVID-19 risk at higher rates than the general population, with 68% indicating practicing average to very high levels of physical distancing.^11^ Any gains in HRQoL associated with reduced psychosocial impacts or behavioral modification may have important implications in terms of the benefits, and therefore of the cost-effectiveness, of PrEP interventions for IC individuals.

A growing body of studies have sought to compare HRQoL before and during the pandemic based on self-reported data from measures like the EQ-5D.^12–17^ Studies across multiple countries have typically demonstrated a deterioration in HRQoL during the pandemic, particularly in dimensions relating to anxiety/depression and ability to carry out usual activities.^12,13^ However, to our knowledge, existing data primarily relate to general population samples or conditions where immunodeficiency may be a factor but is not the primary focus of the study (eg, mixed skin conditions).^17^ HRQoL data collected during periods of government-enforced population-level restrictions could potentially serve as a proxy for the short-term quality of life (utility) loss associated with shielding by individuals with IC conditions early in the pandemic. However, such estimates fail to capture the impact of long-term behavioral modification at a point in time when the general public no longer faces restrictions.

This study aimed to elicit the HRQoL burden associated with behavioral modification to prevent COVID-19 and potential benefits of PrEP for individuals with IC conditions. This was assessed using time trade-off (TTO), EQ-5D-5L, and visual analog scale (VAS) valuation of vignettes by individuals with IC conditions and with members of the general public in the United Kingdom. This study aimed to estimate utility weights that can be used in future economic evaluations of preventative therapies against COVID-19. Any improvements in HRQoL may be contingent on patients changing their behavior following PrEP; therefore, a secondary objective of the study was to explore the anticipated behavioral and emotional impacts of PrEP from a patient perspective.

## METHODS

Health state vignettes were developed to describe the typical impacts of various levels of behavioral modifications to prevent COVID-19 infection for individuals with IC conditions. Beyond the behavioral impacts of shielding to prevent COVID-19, individuals classified as immunosuppressed may have very different HRQoL due to the nature and severity of their underlying health condition. This study aimed to assess the potential benefits of PrEP due to behavioral change. As such, vignettes aimed to isolate the impact of shielding and preventative behaviors irrespective of the IC condition rather than describe and measure the HRQoL impacts of the IC conditions themselves.

**Figure 1.**
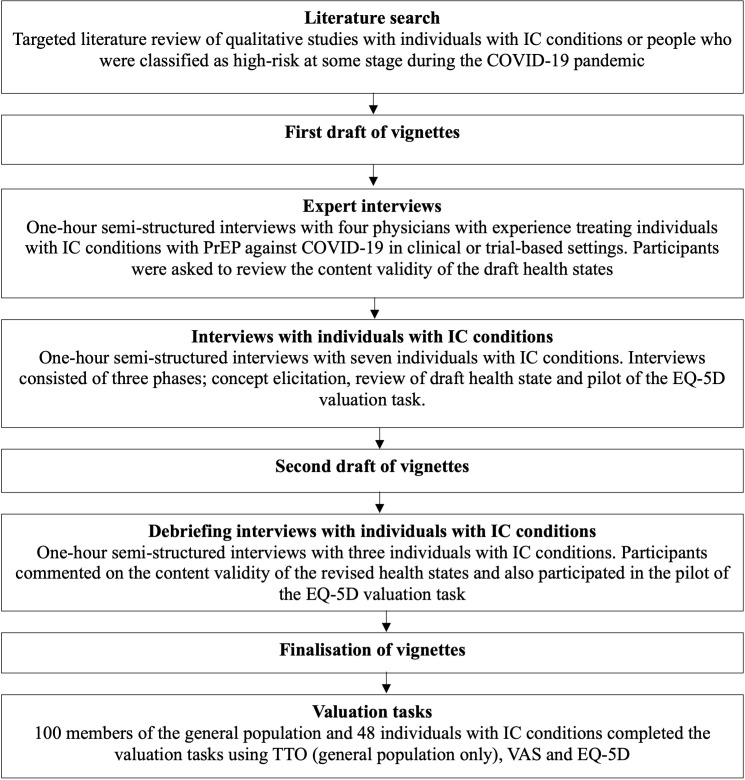
Overview of Methods Abbreviations: IC, immunocompromised; PrEP, pre-exposure prophylaxis; TTO, time trade-off; VAS, visual analog scale.

Prior to data collection, the study protocol was submitted to an independent institutional review board for ethical review and was deemed minimal risk and therefore exempt from full review (WCG IRB #1-1592870-1). An overview of the methods is provided in **Figure 1**.

### Literature Review

To understand the domains of HRQoL impacted by shielding and other behavioral modifications, a targeted literature review was conducted focusing on qualitative studies with individuals with IC conditions or people who were classified as high-risk during the COVID-19 pandemic. The search was performed in MEDLINE and Embase (using Ovid) using search terms relating to “COVID-19,” “preventative behaviors,” “immunocompromised,” and “qualitative.” Search terms are provided in the **Supplementary Material**. Studies were thematically analyzed.

### Health State Development

Draft descriptions of 4 health states were developed based on the literature review:

- **Health state 1 (“shielding”):** A patient who is IC and highest risk and has not received PrEP. They minimize their face-to-face interactions with the public by staying at home as much as reasonably possible.
- **Health state 2 (“semi-shielding”):** A patient who is IC and highest risk and has not received PrEP. They currently modify their behavior to reduce their risk of COVID-19 but do engage in selective activities outside of their home.
- **Health state 3 (“post-PrEP”):** A patient is IC and highest risk but has received PrEP. This additional protection allows them to largely return to their pre-pandemic lifestyle.

An additional health state describing a post-PrEP scenario was designed to be valued by individuals with IC conditions only. The description was designed to be relevant to a broad cross-section of people who are IC with different etiologies and asked patients to imagine that the treatment would provide a level of protection similar to a fully vaccinated immunocompetent individual. This approach was designed based on a previous study that valued the HRQoL impact of immunotherapy for peanut allergy.^18^ This approach was chosen over valuation of the hypothetical shielding and semi-shielding vignettes as it allowed patients to draw directly from their own current experiences.

### Health State Validation and Revision

Semi-structured virtual interviews with 4 clinicians (2 US, 2 UK) and 10 patients were conducted to validate the draft vignettes and finalize their content. Clinicians were identified based on their experience treating patients with PrEP for COVID-19 in routine clinical and/or clinical trial-based settings. Clinicians were presented with the draft vignettes and asked to comment on their accuracy and identify any missing content important to the description of IC individuals’ HRQoL relating to behavioral modifications due to COVID-19.

Patients were recruited via a specialist recruitment agency. To be eligible, patients met the following criteria: (1) were interested in receiving PrEP for COVID-19, (2) had a self-reported diagnosis of a condition classified as a highest-risk clinical subgroup by the UK Department of Health and Social Care or otherwise ineligible for a COVID-19 vaccine based on immune compentence,^19^ (3) were over 18 years old, (4) lived in the UK, (5) were able to read and speak English fluently, and (6) provided informed consent. In practice, individuals who were not interested in receiving PrEP for COVID-19 would not be required to receive such treatments. As such, these individuals were excluded from the study.

Interviews were performed in 2 iterative rounds. In the first round, 7 patients and 4 clinicians provided feedback on the accuracy of the draft health states. Patient interviews also included a concept elicitation section consisting of open- ended questions to understand the HRQoL burden of shielding and behaviors currently and throughout the pandemic. A pilot of the EQ-5D valuation task was also conducted. Information from the first-round interviews was used to refine the accuracy and generalisability of the vignettes. In the second round, 3 patients provided feedback on the revised vignettes, with a focus on any outstanding areas of uncertainty. It was expected that patients and clinicians would have sufficient experience of shielding and preventative behaviors to provide feedback on all health states. The vignettes were finalized following the second round of feedback (see **Supplementary Material**).

### Health State Valuation

Data were collected via online interviews lasting approximately 30 minutes, conducted in November 2022. Prior to all interviews, participants were provided with information about the study and asked to complete a consent form followed by a background questionnaire. Interviews were conducted by 8 moderators using a structured interview guide. All moderators were experienced in vignette valuation interview methods including TTO and EQ-5D valuation and trained on the study procedures. Practice interviews were conducted with the members of the study team and interview procedures were refined prior to final data collection. Structured feedback questions completed by moderators following each interview were used to exclude responses from participants demonstrating poor understanding or concentration during the valuation tasks.

**Patient evaluation**: A target sample size of 50 individuals with IC conditions was sought using the same recruitment methods and inclusion criteria as the vignette development interviews. During the first task, participants were asked to value health states using a VAS ranging from 0 (worst possible health) to 100 (full health). To anchor their scores, participants were first asked to indicate where they would value “dead” on the 0-100 scale. Next, participants were asked to rate their current HRQoL, thinking about any health conditions they experience and any current changes to their behavior due to COVID-19. Following the VAS ratings, participants completed the EQ-5D-5L for their current health. Participants were then presented the post-PrEP health state and asked to think about how they would feel and behave if they received the treatment described in the vignette. They then rated the vignette using the VAS and EQ-5D-5L.

The 10 vignette development interviews were included in the final sample of 50. Valuation procedures piloted during the development interviews differed slightly from the main study. First, the valuation task occurred at the end of the interview, meaning participants had already spoken in-depth about their experience shielding from COVID-19. Second, the post-PrEP health state included slightly different wording (**Supplementary Material**). To assess the appropriateness of pooling responses, a sensitivity analysis excluding the pilot responses was performed.

During the interviews, participants were asked additional open-ended questions to further understand the potential HRQoL benefits of PrEP. Participants who completed the pilot task (n = 10) were not asked about their expected behavioral and emotional impacts of treatment.

**General public**: Adult (≥18 years of age) members of the UK general population were recruited using a convenience sample with quotas aiming to achieve a broadly representative sample according to UK 2021 census data. A target sample size of 100 participants was established based on previous studies of a similar nature.^20–22^

Health states were evaluated using 3 established valuation techniques: VAS, TTO, and EQ-5D-5L index scores. VAS was used primarily for participants to familiarize themselves with the vignettes. Participants were asked to read each vignette in turn and imagine themselves living in the health state described. The valuation exercise started with participants rating each vignette using a VAS ranging from 0 (worst possible health) to 100 (full health).

Next, participants valued the health states using the TTO approach based on the Euroqol cTTO protocol.^23^ The method determined the point of equivalence between 10 years in the vignette health state and X years in “full health.” Interviews used a “ping pong” approach with participants trading months of full health (in 6-month increments) to avoid living in the health state until their trade-offs iteratively narrow to the point of indifference, where the participant believes the 2 health states are equally preferable. If participants reported a preference for zero years of full health (ie, dead) over 10 years in the described health state, a lead-time TTO method was used to indicate how much worse than dead a health state was believed to be. Finally, participants were asked to rate each health state according to the 5 domains of the EQ-5D-5L in line with recent NICE guidance regarding the valuation of vignettes.^24^

Each health state was valued using all 3 methods before moving onto the next health state. A practice state was included to familiarize participants with the different valuation methods and assess participant engagement and understanding prior to the main task. Health states were shown in the order of decreasing levels of restrictive behavior.

### Statistical Analysis

Sociodemographic data and participants’ own EQ-5D-5L scores were summarized descriptively. All utility values were summarized using means and 95% confidence intervals (CI). VAS ratings for each vignette were rescaled such that the value for the dead state was fixed at zero and all other values varied between 100 and the worst health state using the following formula:

$$V^{\prime }=\left(\frac{V-V_{\text{Dead }}}{100-V_{\text{Dead }}}\right)*100$$

where *V′* is the rescaled VAS value, *V* is the original VAS value, and *V^Dead^* is the value given to the “dead” state.

TTO data (general population sample only) were scored according to the point of indifference, assuming full health has a utility weight of 1.0. Index EQ-5D-5L scores for each state were calculated using a mapping function for the EQ-5D-3L reflecting UK preference weights.^25,26^ Analysis was completed using R 4.1.227; the R package eq5d^27^ was used to calculate the EQ-5D-5L utility values. Responses to the open-ended questions relating to behavioral and emotional impacts of PrEP were analyzed thematically.

## RESULTS

### Literature Review

In total, 14 studies were identified, covering a wide range of conditions, including cancer, end-stage renal disease, lupus, and related conditions.^28–41^ The HRQoL impacts of shielding or lifestyle modification could be grouped into 3 general themes: lifestyle impacts, physical health impacts, and psychological impacts.

Lifestyle impacts included social activities, employment and finances, and ability to participate in usual activities. Physical impacts were more varied. Some reported decreased physical activity due to being housebound; in some cases, this also caused a decline in physical health. Others reported no change or even improvements in ability to exercise due to increased time. Psychological impacts were the most frequently reported impact of behavioral modification. Increased levels of anxiety, depression, and feelings of isolation or exclusion were most commonly described. An in-depth summary is provided in the **Supplementary Material**.

### Vignette Development Interviews

Ten individuals with IC conditions, including neurological disorders (n = 5), solid organ cancer (n = 2), and hematological diseases (n = 3) were interviewed to review and refine the health states. Patients had a mean age of 46 years (SD, 14.4; range, 24-73 years) and were mainly female (n = 8).

All patients indicated they had fully shielded at the start of the pandemic, however, only 1 patient was fully shielding at the time of interview. Most patients could be classified as semi-shielding, reporting a cautious approach and continuing with aspects of shielding and social distancing (eg, avoiding busy places, avoiding large social gatherings). Some participants felt they had reduced autonomy over their shielding decisions after the easing of government restrictions. This was because their work or family commitments meant they had no choice but to reengage in usual activities similar to a pre-pandemic level. However, only 1 patient indicated they were no longer modifying their behavior to prevent COVID-19 in any way.

Even those who indicated they had largely returned to pre-pandemic behavior described experiencing continued social impacts because of the pandemic. This included being selective about with whom and where they socialized, often avoiding crowded indoor settings. Some described dependence on virtual communication as a means of feeling connected. Most participants described an ongoing work and/or financial impact as well as a variety of limitations to their usual activities.

Individuals with IC conditions frequently reported increased mental health problems due to their increased risk of COVID-19. Sources of anxiety were wide-ranging and varied depending on the level of preventative behavior patients continued to adopt. For instance, more vigilant and restrictive participants reported anxiety arising from the risk of catching COVID-19 but also social anxiety at the thought of returning to “normal.” One patient had even been diagnosed with agoraphobia resulting from heavily isolating throughout the pandemic. Feelings of depression and low mood were also common among this group. Participants described experiencing increased levels of psychological impacts at times when they were practicing higher levels of physical distancing (eg, when they were required to fully shield).

Individuals with IC conditions also provided feedback on the draft health states alongside input from 4 clinicians with experience administering PrEP to individuals with IC conditions. When reviewing the health states, patients’ previous experiences and current behavior appeared well aligned with the shielding (HS1) or semi-shielding (HS2) health state descriptions. A summary of interview findings, changes to wording, and final health states is provided in the **Supplementary Material**.

### Patient Valuation

In total, 50 individuals with IC conditions completed the valuation tasks; responses from 2 participants were excluded due to insufficient understanding and effort, resulting in a final sample of 48 participants.

Sociodemographic characteristics of the final sample are presented in **Table 1**. The sample contained a wide age range, from 19 to 79 years (mean [SD], 43 [13.7] years), 73% were female, and participants with a range of underlying conditions were included. Three participants reported continuing to fully shield (6%), and 4 participants indicated they were no longer taking any precautions to prevent COVID-19 (8%). Remaining participants indicated they were currently semi-shielding (41/48; 85%). Approximately half (56%) had received a positive COVID-19 test result during the pandemic.

**Table 1.** Sociodemographic Characteristics of the General Population (n = 100) and Individuals with IC (n = 48) Conditions in the UK (Nov.–Dec. 2022)

**Characteristic**		**Individuals with IC Conditions (n = 48)**	**General Population (n = 100)**	**UK Population**
Age (y)	Mean (SD)	45.9 (14.4)	42.8 (13.7)	39.4^a^
	Range	19.0-79.0	21.0-75.0	
Sex (%)	Male	13 (27.1)	49 (49)	49^a^
	Female	35 (72.9)	50 (50)	51^a^
	Other	–	1 (1)	
Ethnicity (%)	White	–	79 (79)	86^a^
	Asian or Asian British	–	11 (11)	8^a^
	Black, African, Caribbean or Black British	–	4 (4)	3^a^
	Mixed/ multiple ethnicity	–	5 (5)	2^a^
	Other	–	1 (1)	1^a^
Current EQ-5D utility	Mean (SD)	0.57 (0.25)	0.84 (0.18)	
	Range	0.50-0.65	0.10-0.99	
	95% CI	-0.07-0.92	0.80-0.88	
Had COVID-19 (%)	Yes (positive test)	27 (56.2)	71 (71)	
	Suspected but not confirmed	2 (4.2)	7 (7)	
	No	16 (33.3)	22 (22)	
	Don”t know	3 (6.2)	0 (0)	
Condition (%)	Rare neurological conditions	6 (12.5)	–	
	Solid organ cancer	12 (25.0)	–	
	Hematological disease	12 (25.0)	–	
	Renal conditions	2 (4.2)	–	
	Liver conditions	4 (8.3)	–	
	Solid organ transplant recipients	2 (4.2)	–	
	Receiving immunosuppressant therapy	7 (14.6)	–	
	Immune deficiency	3 (6.3)	–	
	Any long-term condition	-	33 (33)	36^b^
Current behavior (%)	Shielding	3 (6.3)	-	
	Semi-shielding	41 (85.4)	-	
	No behavior modification	4 (8.3)	-	

^a^Figures based on data from the 2021 UK national census (Office for National Statistics https://www.ons.gov.uk/census).

^b^Figure based on data from the 2013 UK Opinions and Lifestyle Survey (Office for National Statistics). http://www.ons.gov.uk/peoplepopulationandcommunity/healthandsocialcare/healthandlifeexpectancies/compendium/)

Abbreviation: CI, confidence interval.

Patient utility values for current (participants’ own HRQoL at the time of the interview) and post-PrEP health states (their rating of the vignette) estimated using VAS and EQ-5D methods are presented in **Table 2**. Results from the VAS task indicate an increase of 17.4 points (95% CI, 11.12-23.7) between current and post-PrEP HRQoL. Mean utility was expected to increase by 0.082 (95% CI, −0.010-0.175), from a current average of 0.574 (95% CI, 0.503-0.645) to 0.656

(95% CI, 0.596-0.727) in the post-PrEP state. Sensitivity analysis found no difference in estimates between the full sample and when excluding the responses to the slightly different pilot health state (*t*-test nominal *P* value = .630).

**Table 2.** Health State Values of Individuals with Immunocompromised Conditions Using VAS and EQ-5D Methods

**Health State**	**VAS**		**EQ-5D-5L**	
	**Mean**	**95% CI**	**Mean**	**95% CI**
Full sample (N=48)				
1. Current HRQoL	59.1	54.1-64.0	0.574	0.503-0.645
2. Post-PrEP	76.5	72.4-80.5	0.656	0.596-0.717
Utility difference				
Current HRQoL → Post-PrEP	17.4	11.1-23.7	0.082	-0.010-0.175

Abbreviations: CI, confidence interval; HQoL, health-related quality of life; PrEP, pre-exposure prophylaxis; TTO, time trade-off; VAS, visual analog scale.

**Figure 2.**
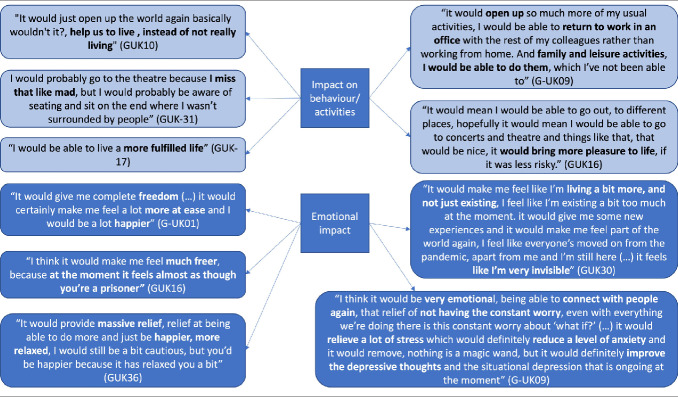
Example Quotations

**Anticipated behavior change:** Participants’ responses relating to expected impact of the treatment described in the vignette on HRQoL domains are summarized in **Figure 2**. These questions were asked to participants only after the pilot interview phase (n = 40). Thirty participants (75%) indicated they would change their behavior if they received the treatment described. The most common types of changes to HRQoL were increased social activity and being able to go to busy places. Participants also reported many anticipated emotional impacts of the treatment; most commonly, participants felt they would feel less anxious.

**Figure 3.**
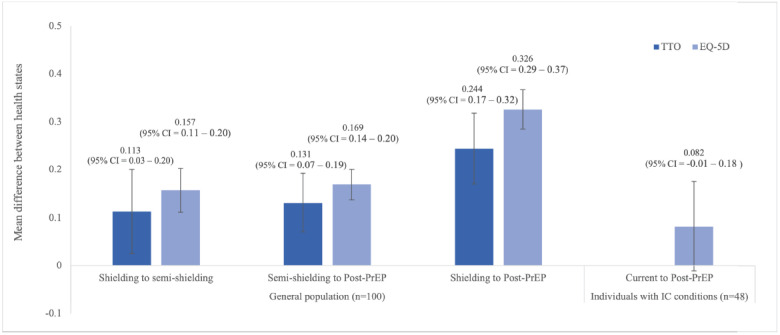
Comparison of Utility Differences Between Health States Based on Population and Estimation Method Abbreviations: CI, confidence interval; IC, immunocompromised; PrEP, pre-exposure prophylaxis; TTO, time trade-off.

**General public valuation:** In total, 101 members of the general public completed the valuation tasks. One participant was excluded due to insufficient understanding of the task based on moderator feedback, leaving a final sample of 100 participants. Participant demographics of the sample were generally comparable to the UK general population according to 2021 census data (**Table 1**).^42^ Utility weight estimates for each health state using VAS, EQ-5D and TTO approaches are shown in **Table 3**. Mean VAS scores for the 3 health states followed the expected order and ranged from 48.4 (shielding) to 74.7 (post-PrEP). TTO weights for the health state vignettes ranged from 0.662 (shielding) to 0.906 (post-PrEP). Differences in utility between health states were also estimated (**Figure 3**). Moving from semi-shielding to the post-PrEP health state (ie, little to no behavioral modification) resulted in a utility gain of 0.131 (95% CI, 0.070-0.192).

**Table 3.** General Population Health State Values Using VAS, EQ-5D and TTO Methods

**Health State**	**VAS**		**EQ-5D-5L**		**TTO**	
	**Mean**	**95% CI**	**Mean**	**95% CI**	**Mean**	**95% CI**
1. Shielding	48.4	44.5-52.3	0.606	0.569-0.643	0.662	0.594-0.731
2. Semi-shielding	53.2	49.4-57.1	0.763	0.738-0.789	0.775	0.721-0.829
3. Post-PrEP	74.7	71.8-77.6	0.932	0.914-0.950	0.906	0.878-0.935

Abbreviations: CI, confidence interval; TTO, time trade-off; VAS, visual analog scale.

Public valuation of health states using the EQ-5D-5L resulted in utility scores ranging from 0.606 (shielding) to 0.932 (post-PrEP). The estimated gain in utility associated with an improvement from shielding to post-PrEP was 0.326 (95% CI, 0.285-0.367) and from semi-shielding to post-PrEP was 0.169 (95% CI, 0.138-0.200).

## DISCUSSION

This study elicited utility gain associated with PrEP against COVID-19 for individuals with IC conditions using multiple vignette valuation techniques. Data were collected from both a patient and a general population sample to improve the validity of findings. The development of the vignettes adopted a multimethod approach combining different data sources across iterative rounds of review to better support content validity.

Health state vignettes focused on the behavioral and emotional impacts of behavior modification (eg, shielding) rather than the underlying IC conditions. Variation in the severity and disease burden of immunosuppressive conditions means it is not feasible to draw conclusions from the absolute values of each health state. Instead, interpretation should focus on the relative utility change between health states. When considering relative utility change, valuations using the different methods across the 2 populations differ slightly; however, they are similar in magnitude and order. The patient valuations resulted in an average utility difference of 0.082 between current HRQoL and post-PrEP HRQoL. The values from the general population resulted in larger estimated HRQoL benefits of PrEP. When valued by a general population sample, the mean utility gain from moving from fully shielding to post-PrEP was estimated to be between 0.244 (TTO valuation) and 0.326 (EQ-5D-5L valuation). Values from the general public sample indicated the utility gain from semi- shielding to post-PrEP health state was between 0.131 (TTO) and 0.169 (EQ-5D-5L) depending on the valuation approach.

The study also demonstrated the potential behavioral and emotional benefits of PrEP for prevention of COVID-19 through open-ended interview questions with a subset of individuals with IC conditions (n = 40). Results confirmed the perceived benefit of prophylaxis across this population with 75% of participants indicating they would change their behavior if they had access to the PrEP described in the vignette. Crucially, almost all patients (37/40) felt that PrEP would offer an emotional benefit. This finding demonstrates the potential emotional benefit of PrEP to all individuals with IC conditions, regardless of the level of behavioral restrictions they currently place on their day-to-day life. This finding is in line with previous studies that frequently report increased levels of anxiety and depression as a result of shielding from COVID-19.^28–31,33,34,36–40^ In particular, Lasseter et al found 72% of clinically extremely vulnerable patients reported increased levels of anxiety since beginning to shield/make other restrictions as measured by the Generalised Anxiety Disorder Assessment.^34^

To our knowledge, this is the first study to estimate the potential HRQoL benefits of the psychosocial and behavioral impacts of PrEP for COVID-19. This study also adds additional insights regarding the impact of behavioral changes necessitated by the threat of COVID-19 and the knock-on effects in terms of HRQoL.^12–16^ Previous studies typically focus on the impacts of the pandemic more generally or government-mandated restrictions at the height of the pandemic from a general population perspective. Nonetheless, findings across studies are similar in demonstrating a HRQoL detriment associated with the pandemic, primarily driven by an increase in the anxiety and depression and reduced ability to carry out usual activities.

Some previous research has utilised EQ-5D data collected as part of longitudinal studies initiated prior to the pandemic to understand the subsequent impact of COVID-19.^12–15^ Alternative studies were designed specifically to investigate the impacts of COVID-19 meaning pre-pandemic estimates of HRQoL are reliant on retrospective data collection.^16^ While comparable, previously estimated utility decrements attributable to COVID-19 are typically smaller than the levels observed in this current study. For example, a recently published cross-sectional study estimated an average decrement of 0.053 (SD, 0.030-0.076) for the UK general population based on data collected in late 2020.^16^ The higher utility decrements estimated within this study may be due to several factors. In particular, the prolonged nature of the behavioral modification and social isolation are unique to this study. Additionally, continued lack of sufficient protection against COVID-19 and increased anxiety due to the high-risk conditions of the population of interest may lead to differences.

The results of this study should be interpreted in the context of some limitations. The vignette descriptions were developed for this study following recommended methods for health state vignette development, using information from a literature review followed by interviews with patients and clinicians.^43^ Patients and clinicians reviewed the draft vignettes and provided feedback to improve the content and face validity; however, public valuation using vignettes leads to an inevitable simplification in the description of the health state. As with any condition, the evidence reviewed when developing the vignettes revealed a degree of variability in patient’s experiences, with people adopting varying combinations of preventative behaviors to better protect themselves against COVID-19. Health state vignettes aimed to capture the average experience of all patients but in doing so may overlook some nuances on patient behavior that may be of varying importance to those asked to value the vignettes. In this study, differences between health states included improvements in anxiety and depression. These statements were included based on feedback from individuals with IC conditions and are further supported by qualitative descriptions of improved psychological status in this study. Nonetheless, the assumption that anxiety and depression would be completely alleviated by PrEP may be an oversimplification. However, the entire UK public has some experience of lockdown and the need for shielding. The ability of the general population to draw on their own experiences of the COVID-19 pandemic may therefore mitigate the impact of variability between individual experiences not captured within vignettes.

Furthermore, the health states were presented to participants in the order of decreasing levels of restrictive behavior, rather than randomly, to limit the vignettes” complexity and decrease potential confusion for the participant. However, this approach may result in the potential for some bias in participants responses.

The patient valuation task provided an additional approach which allowed patients to form their judgements based on their direct experiences. However, the sample size of the patient population is a further limitation of the study. A larger sample may have produced more generalisable data. Crucially, the post-PrEP health state assumed recipients would receive a level of protection similar to fully vaccinated immunocompetent individuals; this assumption should be treated with caution due to continued evolution of new COVID-19 strains. Further research is required to understand the expected HRQoL impacts and behavioral and emotional response of patients where levels of protection may be uncertain. In addition, the potential bias due to the inclusion criteria should be considered, participants were required to be interested in receiving PrEP, and therefore may be expected to view the treatment favorably. Despite the potential limitations, this study is the first of its kind to estimate the utility impacts of PrEP for COVID-19 in individuals with IC conditions. The outputs of this study quantify the psycho-social impact of increased risk of COVID-19 in individuals with IC conditions which should be considered as part any future health economic evaluations of PrEP therapies.

## CONCLUSIONS

This study provided utility values demonstrating the HRQoL benefit associated with reduced psychosocial impacts and behavioral restriction associated with PrEP for prevention of COVID-19 for those who are IC and therefore at increased risk of serious infection. Estimates were derived from both a patient and a general population sample based in the UK. Prevention with PrEP was estimated to result in a utility gain between 0.082 and 0.326 dependent on valuation approach and expected change in behavioral restrictions. The utility values estimated in this study could enable future cost-effectiveness assessments of PrEP for COVID-19 to consider the behavioral and emotional benefits of PrEP.

## Supplementary Material

Online Supplementary Material
