# Supplementary material for: Estimating the Health-Related Quality of Life Benefit of Prophylactic Treatment for COVID-19 in Immunocompromised People: A Multimethod Valuation Study
Source: J Health Econ Outcomes Res. 2024 Jul 24;11(2):20–8. doi: 10.36469/001c.120605 (PMC11276479; doi:10.36469/001c.120605)
Supplement: Online Supplementary Material [file jheor_2024_11_2_120605_238239.pdf]

Journal of Health Economics and Outcomes Research

Online Supplementary Material

Estimating the Health-Related Quality of Life Benefit of Prophylactic Treatment for COVID-19 in Immunocompromised People: A Multimethod Valuation Study. *JHEOR*. 2024;11(2):20-28. doi:10.36469/jheor.2024.120605

Supplementary Material 1: Literature Review Wording

Supplementary Material 2: Summary of Targeted Literature Search Used to Develop Draft Vignettes

Supplementary Material 3: Interview Findings

Supplementary Material 4: Final Health States

This supplementary material has been provided by the authors to give readers additional information about their work.

This is an open-access article distributed under the terms of the Creative Commons Attribution 4.0 International License (CCBY-4.0). View this license's legal deed at http://creativecommons.org/licenses/by/4.0 and legal code at http://creativecommons.org/licenses/by/4.0/legalcode for more information.

SUPPLEMENTARY MATERIAL 1. LITERATURE REVIEW WORDING

	Concept	Search Terms	Results	Comments
1	**IC population**	(immunosuppress* or immunocomp* or immunodeficien* or deficien* or autoimmun* or HIV or transplant* or cancer* or malignan* or tumo?r* or leuk?emia or oncol* or dialysis or h?em* or rheumat* or malnutrition or high risk or vulnerable).ti,ab.	16,219,461	Adapted from: Parker, E.P., Desai, S., Marti, M., Nohynek, H., Kaslow, D.C., Kochhar, S., O'Brien, K.L., Hombach, J., Wilder-Smith, A. Response to additional COVID-19 vaccine doses in people who are immunocompromised: a rapid review. *Lancet Global Health*.2022 10(3):e326-e328.<br><br>Additional words added: high risk, vulnerable
2	**Social restrictions**	(social isolat* or patient isolat* or emotional isolat* or quarantine or social distanc* or social support or lonel* or aloneness or solitude or shield or shielding or bubble).ti,ab.	273,730	Adapted from: Williams, C.Y., Townson, A.T., Kapur, M., Ferreira, A.F., Nunn, R., Galante, J., Phillips, V., Gentry, S. and Usher-Smith, J.A., 2021. Interventions to reduce social isolation and loneliness during COVID-19 physical distancing measures: A rapid systematic review. *PloS One*, 16(2), p.e0247139.<br><br>Additional words added: shield, shielding, bubble
3	**Qualitative studies**	(interview* or qualitative or focus group* or lived experience* or conceptual model* or narrative* or mixed method*).ti,ab,kw.	1,566,271	
4	**Covid-19**	exp coronavirus/	243,932	NICE search filter: (https://www.nice.org.uk/process/pmg35/chapter/appendix-search-strategy-for-medline-ovid-platform).
5		((corona* or corono*) adj1 (virus* or viral* or virinae*)).ti,ab,kw.	9,997	
6		(coronavirus* or coronovirus* or coronavirinae* or Coronavirus* or Coronovirus* or Wuhan* or Hubei* or Huanan or "2019-nCoV" or 2019nCoV or nCoV2019 or "nCoV-2019" or "COVID-19" or COVID19 or "CORVID-19" or CORVID19 or "WN-CoV" or WNCoV or "HCoV-19" or HCoV19 or CoV or "2019 novel*" or Ncov or "n-cov" or "SARS-CoV-2" or "SARSCoV-2" or "SARSCoV2" or "SARS-CoV2" or SARSCov19 or "SARS-Cov19" or "SARSCov-19" or "SARS-Cov-19" or Ncovor or Ncorona* or Ncorono* or NcovWuhan* or NcovHubei* or NcovChina* or NcovChinese*).ti,ab,kw	622,756	
7		(((respiratory* adj2 (symptom* or disease* or illness* or condition*)) or "seafood market*" or "food market*") adj10 (Wuhan* or Hubei* or China* or Chinese* or Huanan*)).ti,ab,kw.	1,934	
8		((outbreak* or wildlife* or pandemic* or epidemic*) adj1 (China* or Chinese* or Huanan*)).ti,ab,kw.	812	
9		"severe acute respiratory syndrome*".ti,ab,kw.	71,749	
10		or/4-9	646,118	
11		1 and 2 and 3 and 10	940	
12		limit 11 to yr="2019 -Current"	926	
13		limit 12 to humans	780	
14		remove duplicates from 13	507	

SUPPLEMENTARY MATERIAL 2: SUMMARY OF TARGETED LITERATURE SEARCH USED TO DEVELOP DRAFT VIGNETTES

Fourteen studies were identified during the targeted literature review.[13-26] Studies covered a wide range of conditions. Several studies related to the experiences of IC people (eg, patients undergoing dialysis for end-stage kidney disease). However, due to the emerging nature of evidence, the review criteria was extended to also include qualitative studies in populations previously considered clinically extremely vulnerable and advised to shield during the early wave on the pandemic (eg, asthma, COPD). Differences in populations and the timing of studies meant behavioral modifications ranged from shielding, government-enforced lockdowns, and protective behaviors short of shielding.

Lifestyle Impacts

Lifestyle impacts included social activities, employment and finances and ability to participate in usual activities. For many, restrictions in socializing were the least manageable part of shielding.[28] Participants reported reductions in their social circle and a switch to virtual means of communication.[26,27,30,33,35] Individuals who were able to work from home typically described receiving good support from their employer to enable prolonged remote working.[32,35] However, individuals with customer facing roles or busy work environments described being unable to continue working.[25,30,31,34] This often led to financial problems which contributed to the mental health impacts of shielding.[30,31,34] Shielding and other behavioral modifications led to a sudden change in many aspects of patients' usual routines. People were no longer able to participate in usual activities including going to the gym, church, activity groups[25,27,30,34,35] and were often reliant on online shopping or help from others to receive groceries.[24,31,32,34,35] Behavioral modifications had little-to-no impact on daily activities within the home such as housework.[27]

Physical Impacts

The physical impacts of behavioral changes to prevent COVID-19 were commonly reported but varied across interview participants. While some reported decreased physical activity due to being housebound, others reported increased physical activity due to increased leisure time as a result of the pandemic. As a result of reduced physical activity and an inability to attend physio appointments face-to-face, some respondents reported increased "stiffness," "pain," "soreness," and breathing difficulties.[23,27,28] However, deterioration in physical health was largely dependent on the pre-existing condition of each individual; for instance, those suffering from musculoskeletal conditions reported issues more frequently.[22–24,27]

Self-Care Impacts

No evidence was found to suggest any form of preventative behavioral modifications impacted the ability to complete self-care activities such as washing and dressing.

Psychological Impacts

Psychological impacts were the most frequently reported impact across all studies. Respondents in all studies reported increased levels of anxiety since before the pandemic. Sources of anxiety were varied but included fear related to their high-risk status but also anxiety about returning to their pre-pandemic behavior and around the actions of the general public as government-mandated measures began to be lifted (eg, social distancing, mask wearing).

Depression or "low mood" were also commonly reported. Individuals most frequently linked their depression to prolonged social isolation and inactivity.[22,24,27,28,30–34] For many, this resulted in a cyclical relationship whereby their depression led to reduced enjoyment and motivation to do usual activities such as exercise or work, and avoidance of social interactions [24,27]. For others, depression was linked to more tangible causes such as employment or financial issues.[25,30,31,34] Depression and anxiety were often experienced simultaneously and the impacts of the two experiences were often inseparable. Feelings of anxiety and depression increased as the need for shielding continued. Loneliness and isolation and feelings of being left behind or excluded as a result of shielding or modifying behavior were also frequently reported across studies.

SUPPLEMENTARY MATERIAL 3: INTERVIEW FINDINGS

Draft Content	Interview Feedback	Change	Final Content
**1: Shielding: Patient is immunocompromised and "highest-risk" and not treated with a prophylactic**			
You have a **long-term health problem** that has **affected your immune system** which means you are **at risk of catching infections**		Changed to include consequences of infections	You have a **long-term health problem** that has **affected your immune system** which means you are **at risk of catching infections and becoming seriously ill**
You have **not** received a treatment to protect you from getting COVID-19	**HCP2**: People in this group would definitely opt for vaccine but would not be confident about the level of protection<br>**HCP4**: Most are likely to have been vaccinated but think/have been told that vaccine is effective for them<br>**UK04:** Interpreted "no treatment" as no vaccination	Reworded to removed ambiguity about what is meant by "treatment"	**You may have been vaccinated but are still at risk of COVID-19**
You socialise **virtually** (over the phone or virtual calls)	**UK02:** Would visit parents through window<br>**UK04:** accurate at start of pandemic. Now meets people in garden, socially distanced<br>**UK08:** Formed bubble with adult children and grandchildren<br>**UK10:** Formed bubble with aunt and cousin	Changed to include limited in-person interactions and reflect reduction in virtual socialisation at this stage in pandemic	You socialise with very close friends and family outdoors and at a safe distance.

Draft Content	Interview Feedback	Change	Final Content
	**HCP1:** accurate for some but others will socialize from a distance wearing a mask<br>**UK01:** virtual socialisation less frequently than at start of pandemic<br>**HCP4:** virtual socialisation less common now. Qualifier recommended (“sometimes/occasionally”) or socialization limited to very close friends/family		
You **avoid** physical contact with friends and family (eg, hugs)	**UK02:** accurate<br>**UK04:** accurate<br>**UK10:** accurate even within bubble<br>**UK08:** accurate but not within bubble<br>**HCP4:** suggested qualifier: “mostly”	No change	You **avoid** physical contact with friends and family (eg, hugs)
You **are able to** do your usual activities such as housework, however you need to get your grocery shopping online or rely on others for help	**HCP2:** Some leave the house for essential shopping if unable to use the internet but would wear a mask and would experience anxiety<br>**UK01:** Accurate<br>**UK08:** Accurate	No change	You **are able to** do your usual activities such as housework, however you need to get your grocery shopping online or rely on others for help
You **are able to** work but only from home	**UK02:** Accurate<br>**UK04:** Accurate – could not do job from home so had to take early retirement<br>**UK06:** Accurate<br>**UK07:** Accurate<br>Retired/unable to work (**UK01, UK08, UK10**)	No change	You **are able to** work but only from home

Draft Content	Interview Feedback	Change	Final Content
You **are able to** exercise, however only at home	**UK08:** Went on walks at quiet times<br>**UK02:** Continued cycling outdoors while shielding<br>**HCP4:** Many restricted by underlying condition	Updated to include outdoor exercise when it's quiet	You are **able to** exercise at home or outdoors during quiet times of day
You **do not** experience any pain	**HCP1, HCP2, HCP3:** sought clarification that this referred to physical pain exclusively<br>**UK08:** sought clarification that this referred to physical pain exclusively. Confused why physical pain might be impacted	Physical pain specified	You **do not** experience any physical pain
You **are able to** wash and dress yourself	**All:** accurate<br>**UK07, UK08:** Confused why this was included/why shielding would impact ability to wash/dress	No change	You **are able to** wash and dress yourself
	**HCP1:** People who fit this profile will likely experience some anxiety<br>**HCP2:** Decline in mental health missing. Typical pattern in this population is feelings of missing out/social isolation, low mood/depression<br>**HCP3:** Loneliness, fear and anxiety<br>**UK01:** Felt like life is passing you by/being left behind<br>**UK08:** Isolating, lonely<br>**UK10:** Lonely	Addition	You often feel anxious or depressed<br>You often feel lonely and isolated
	**UK01, UK06, UK07, UK08, UK10:** All mentioned avoiding public transport	Addition	You avoid public transport

Draft Content	Interview Feedback	Change	Final Content
	**HP2:** Some leave the house for essential shopping if unable to use the internet but would wear a mask and would experience anxiety<br>**HCP1:** accurate for some but others will socialize from a distance wearing a mask	Addition	You wear a mask in indoor and outdoor public areas
**2: Some restrictions: Patient is immunocompromised and "highest risk" and not treated with a prophylactic but does engage in some social activities**			
You have a **long-term health problem** that has **affected your immune system** which means you are **at risk of catching infections**		See HS1	
You have **not** received a treatment to protect you from getting COVID-19		See HS1	
You socialise in person **only** with close friends and family	**HCP1/HCP2/HCP3/HCP4:** Accurate<br>**UK01/UK05:** Accurate<br>**UK04:** Accurate but still only in outdoor settings<br>**UK10/UK02:** Sees more people but still act with caution by keeping distance/balancing risks<br>**UK08:** less accurate – willing to see more people	No change	You socialise in person **only** with close friends and family

Draft Content	Interview Feedback	Change	Final Content
You are **excluded** from some activities that your peers attend	**HCP1/HCP2/HCP3/HCP4:** Accurate<br>UK01/ UK04/UK10: Accurate<br>**UK07/UK01:** Focused on "exclusion" aspect. Don't feel excluded but choose not to attend certain activities	Updated to more patient-friendly phrasing	You are **excluded** from some activities that others attend
You are **able to do** your usual activities such as housework and grocery shopping	**HCP2:** Grocery shopping is key activity that causes fear<br>**UK02/UK06/UK07:** Still avoiding shops, using online shopping where possible<br>**UK04/ HCP3:** Goes shopping but avoids busy shops/times of day<br>**UK10/UK08:** Accurate	Added wording to specify there are still a degree of restriction to activity	You are **able to do** your usual activities such as housework and grocery shopping but avoid busy times of day
You are **able to** work but only from home or in a quiet environment	**UK06:** Accurate – returned to office but switched jobs to be able to work from home more. Wears mask in office<br>**UK07:** Accurate- still primarily works from home with occasional visits to office<br>**UK04:** Accurate- took early retirement due to public-facing job<br>Retired/unable to work (**UK01, UK08, UK10**)	No change	You are **able to** work but only from home or in a quiet environment
You are **able to** exercise at home or outdoors	**UK01/ UK02/UK04/UK10**: Accurate<br>**HCP3:** Some may go to the gym but would go at times they knew it would be quiet<br>**HCP1/HCP4:** Accurate	No change	You are **able to** exercise at home or outdoors
You **do not** experience any pain		See HS1	
You **are able** to wash and dress yourself		See HS1	

Draft Content	Interview Feedback	Change	Final Content
	**UK01/UK02/UK04/UK06/UK07/UK08 :** Avoiding crowded areas/events (eg, concerts, pubs, weddings)<br>**HCP1/HCP3/HCP4:** Avoiding large indoor events/crowded areas	Addition	You avoid crowded events and areas
	**HCP2**: Fear/anxiety (increased use of anxiety and depression medications in this cohort)<br>**HCP1/HCP3/ HCP4**: Emotional impacts are less than in HS1 but fear/anxiety around COVID remains<br>**UK01:** Always thinking about risk of infection<br>**UK04:** Anxiety/panic/paranoid about behavior of the public and social distancing<br>**UK07:** Anxiety about going to the hospital remains	Addition	You sometimes feel<br>anxious or depressed<br>You sometimes feel lonely and isolated
	**HCP1:** Mask wearing is missing for activities such as grocery shopping or exercising outside. Unlikely to be worn around close friends and family<br>**HCP4**: This group are still wearing masks in public spaces – feeling self-conscious about doing this / angry at others for not continuing to wear mask<br>**UK01/UK06/UK07/ UK08/UK10**– wear a mask in public	Addition – non-prescriptive language use as mask-wearing is not universal across this group	You **may choose** to wear a mask in indoor public areas
	**UK01, UK06, UK07, UK08, UK10:** All mentioned avoiding public transport	Addition	You avoid public transport at busy times of the day

Draft Content	Interview Feedback	Change	Final Content
**3: Post-treatment: Patient is immunocompromised and "highest risk" but has received prophylactic treatment**			
You have a **long-term health problem** that has **affected your immune system** which means you are **at risk of catching infections**		See HS1	
**You have received a treatment that gives you a level of protection from COVID-19 which is similar to that given by vaccination in individuals who have a healthy immune system**	**UK01/ UK02/UK06** – required clarification that treatment was different and could be used in addition to vaccine	Additional wording added to clarify treatment is different to vaccine.	**You have received a treatment that protects you from COVID-19. This is an additional treatment to any vaccines you may have received. You now have a level of protection from COVID-19 which is similar to that given by vaccination in individuals who have a healthy immune system**
You **feel able** to socialise with friends and family	**UK07/UK10/UK01/UK04:** Accurate<br>**HCP1/HCP2/HCP3/HCP4:** Accurate	No change	You **feel able** to socialise with friends and family
You are **not excluded** from some activities that your peers attend	**HCP1:** "Some activities" means description is very similar to HS2. Suggested change to "most"<br>**UK04/UK10/UK01/UK07:** Accurate	Wording changed due to ambiguity	You are **not excluded** from most activities that others attend

Draft Content	Interview Feedback	Change	Final Content
	(events such as going to the pub, concerts and cinema mentioned)<br>**HCP1/HCP2/HCP3/HCP4:** Mainly accurate but most still apply a degree of restriction mainly avoiding crowded indoor events.<br>**UK02/ UK04:** Would still avoid crowded places	Additional line added to capture avoidance of busy events	You usually avoid very crowded indoor events
You **are able** to work	**HCP1:** Description is vague. Suggested addition "in person/ outside the home"<br>**UK02:** Did not detect any difference between HS2 and HS3	Additional description added	You **are able** to work outside of the home
You **are able** to exercise	**HCP1:** Description is vague. Suggested addition "outside/in the gym/in the presence of others"<br>**UK02:** Did not detect any difference between HS2 and HS3	Additional description added	You **are able** to exercise in any public indoor and outdoor areas
You do not experience any pain		See HS1	
You are able to wash and dress yourself		See HS1	
	**HCP1:** Comfort/reduced anxiety is big aspect of Evusheld benefits<br>**HCP3:** Relief of anxiety was common pattern throughout the trial. People felt safer<br>**HCP4:** Psychologically in a better place – happier, less anxious, confident<br>**UK01/UK04/UK08/UK10:** Relief in anxiety/ happier/more fulfilled/less restricted	Addition	You do not feel anxious or depressed<br><br>You do not feel lonely or isolated

Draft Content	Interview Feedback	Change	Final Content
	**N/A**	Addition – added to mirror HS1 and HS2	You **are able** to do your usual activities such as housework and grocery shopping

SUPPLEMENTARY MATERIAL 4: FINAL HEALTH STATES

To aid completion of the valuation tasks and prevent respondents making their own inferences, health states aimed to describe all domains of the EQ-5D even where qualitative evidence suggested preventative behavior had little to no impact (eg, self-care).

General public valuation task

Health state 1– Shielding: Patient is immunocompromised and “highest-risk” and not treated with a prophylactic*

- You have a **long-term health problem** that has **affected your immune system** which means you are **at risk of catching infections and becoming seriously ill**
- **You may have been vaccinated but are still at risk of COVID-19**
- You socialise in person with very close friends and family outdoors and at a safe distance.
- You **avoid** physical contact with friends and family (eg, hugs)
- You **are able to** do your usual activities such as housework, however you need to get your grocery shopping online or rely on others for help
- You **are able to** work but only from home
- You **are able to** exercise at home or outdoors during quiet times of day
- You **do not** experience any physical pain
- You **are able to** wash and dress yourself
- You **avoid** public transport
- You wear a mask in indoor and outdoor public areas
- You **often** feel anxious or depressed
- You **often** feel lonely and isolated

*Participants did not see the health state title.

Health state 2 – Semi-shielding: Patient is immunocompromised and "highest risk" and not treated with a prophylactic but does engage in some social activities*

- You have a **long-term health problem** that has **affected your immune system** which means you are **at risk of catching infections and becoming seriously ill**
- **You may have been vaccinated but are still at risk of COVID-19**
- You socialise in person **only** with close friends and family
- You are **excluded** from some activities that others attend
- You are **able to do** your usual activities such as housework and grocery shopping but avoid busy times of day
- You are **able to** work but only from home or in a quiet environment
- You are **able to** exercise at home or outdoors
- You **do not** experience any physical pain
- You **are able** to wash and dress yourself
- You **avoid** crowded events and areas
- You **may choose** to wear a mask in indoor public areas
- You **avoid** public transport at busy times of day
- You **sometimes** feel anxious or depressed
- You **sometimes** feel lonely and isolated

*participants did not see the health state title

Health state 3 – post-treatment: Patient is immunocompromised and "highest risk" but has received prophylactic treatment*

- You have a **long-term health problem** that has **affected your immune system** which means you are **at risk of catching infections**
- **You have received a treatment that protects you from COVID-19. This is an additional treatment to any vaccines you may have received. You now have a level of protection from COVID-19 which is similar to that given by vaccination in individuals who have a healthy immune system**
- You **feel able** to socialise with friends and family
- You are **not excluded** from most activities that others attend
- You usually avoid very crowded indoor events
- You **are able** to do your usual activities such as housework and grocery shopping
- You **are able** to work outside of the home
- You **are able** to exercise in any public indoor and outdoor areas
- You do not experience any pain
- You are able to wash and dress yourself
- You **do not** feel anxious or depressed
- You **do not** feel lonely and isolated

*Participants did not see the health state title.

IC Patient valuation task

Pilot post-treatment health state (completed by 10 participants):

- You have received a treatment that gives you a level of protection from COVID-19 which is similar to that given by vaccination in individuals who have a healthy immune system.
- As your risk of COVID-19 infection is reduced, you can return to your pre-pandemic behavior.
- Your underlying health condition is not affected.

Final post-treatment health state (completed by 40 participants):

- You have received a treatment that protects you from COVID-19. This is an additional treatment to any vaccines you may have received. You now have a level of protection from COVID-19 which is similar to that given by vaccination in individuals who have a healthy immune system.
- As your risk of COVID-19 infection is reduced, you no longer need to modify your behavior to protect yourself from COVID-19.
- Your underlying health condition is not affected.
